# A Comprehensive System for Monitoring Urban Accessibility in Smart Cities

**DOI:** 10.3390/s17081834

**Published:** 2017-08-09

**Authors:** Higinio Mora, Virgilio Gilart-Iglesias, Raquel Pérez-del Hoyo, María Dolores Andújar-Montoya

**Affiliations:** 1 Specialized Processor Architecture-Laboratory, Department of Computer Science Technology and Computation, University of Alicante, 03690 Alicante, Spain; 2 Department of Computer Science Technology and Computation, University of Alicante, 03690 Alicante, Spain; vgilart@ua.es; 3 Department of Building Sciences and Urbanism, University of Alicante, 03690 Alicante, Spain; perezdelhoyo@ua.es (R.P.-d.H.); lola.andujar@ua.es (M.D.A.-M.)

**Keywords:** Smart City, RFID, smart sensor network, urban planning, SOA, Internet of Things, urban accessibility

## Abstract

The present work discusses the possibilities offered by the evolution of Information and Communication Technologies with the aim of designing a system to dynamically obtain knowledge of accessibility issues in urban environments. This system is facilitated by technology to analyse the urban user experience and movement accessibility, which enabling accurate identification of urban barriers and monitoring its effectiveness over time. Therefore, the main purpose of the system is to meet the real needs and requirements of people with movement disabilities. The information obtained can be provided as a support service for decision-making to be used by city government, institutions, researchers, professionals and other individuals of society in general to improve the liveability and quality of the lives of citizens. The proposed system is a means of social awareness that makes the most vulnerable groups of citizens visible by involving them as active participants. To perform and implement the system, the latest communication and positioning technologies for smart sensing have been used, as well as the cloud computing paradigm. Finally, to validate the proposal, a case study has been presented using the university environment as a pre-deployment step in urban environments.

## 1. Introduction

The continuous development of Information and Communication Technology (ICT) has a high impact in different fields of human society, contributing to development through the proposal of new approaches that solve existing problems, such as human-computer interaction, social development, sustainability, and city planning [1]. The growing body of knowledge regarding ICT allows us to propose solutions that exploit the full potential of technological advances. This fact enables organizations to manage social, industrial and business processes in a different manner to increase efficiency and user satisfaction.

Currently, some of these proposals can be seen through emerging concepts such as Smart Cities, Sustainable Cities and Smart Grids, where it is possible to deploy ubiquitous sensor networks for acquiring distributed data and generating valuable information used to improve citizens’ quality of life. In this way, the new ICT innovations drive us towards a *Smart World* concept as a global framework for the collaboration between these smart systems, where different stakeholders will exploit the information provided to achieve their specific purposes according to Smart World’s goals [2].

This idea has been increasing in significance in political agendas and public services. The most representative example is the intensive promotion of Smart City initiatives [3,4]. Cities are complex systems typified by massive numbers of interconnected citizens and companies and numerous modes of transport and communication networks, including services and utilities. The Smart City concept helps municipalities face new challenges due to population growth, such as resource consumption, urban design and design of communication routes [5,6]. They can be addressed with solutions that leverage the full potential that technological development offers. Several major transitions in technology—each one of them important in its own scope—are combining to make the Internet of Everything possible. These proposals use new computing, sensing and telecommunication technologies to provide knowledge and intelligence to the city. In general, the goal is to make better use of resources. The convergence and maturity experienced by previous technologies allow for deployment of applications in the Smart City context to generate knowledge for the management of urban processes. Additionally, they improve efficiency in the design of policies for sustainable resource management and public accessibility [7,8].

Despite the growth of this field, there are still many issues to resolve. One of these issues is focused on achieving Accessible Cities. Accessibility is an element of life quality that is of universal interest, a right of all citizens, a determining factor of the liveability of cities, and an essential element in modern society. It provides security, comfort and autonomy to pedestrians, cyclists, public transport and private motor vehicle users, in its rational use. It is respectful of the urban fabric and should be approached from the many aspects that affect accessibility. In this case, our research focuses on the field of pedestrian mobility, particularly in the most vulnerable group of people with disability. It deals with the study of urban accessibility—urban streets and urban public open spaces—as a means to enhance their participation and contribution in all aspects of social life.

Unfortunately, some spaces of the public built environment are not accessible enough. Pedestrian crossings with curb ramps on only one end, excessively narrow sidewalks and those occupied by street furniture, unsuitable slopes, lack of rest areas over excessively long distances or streets to ensure a leisurely pace, and a lack of markers or clear organization of different traffic flows are just some examples. Its design does not take into account the requirements of people with mobility difficulties and other physical or sensory limitations (of understanding, communication or perception). Much remains to be done to achieve barrier-free areas [9].

In addition, one of the main current problems is the difficulty of knowing what the accessibility deficiencies of cities are. In many cases, regulations are only applied, without checking if they are effective or if there is a degradation that makes the results unusable after a short period of time. For example, consider the construction of a ramp on a street sidewalk that is followed by the installation of an element of street furniture that prevents its use. There is no ongoing analysis over time that confirms that actions are effective or that identifies if they have degraded. The current techniques are focused on conducting biased manual surveys, in very specific environments and time periods, with high cost and without guarantees that these actions are an improvement for the citizens.

For this reason, the main objective of this research is to design a model for evaluating the effectiveness of urban accessibility, improving the habitability of cities and citizens’ quality of life, and achieving equal opportunities for people with disabilities. This model provides automatic monitoring tools supported by technology to dynamically discover, assess and classify urban accessibility issues, generating value-added information regarding accessibility.

To achieve these aims, the starting hypothesis of this research is based on new technologies to yield opportunities for this dynamic and automatic analysis of urban accessibility and overcome difficulties in the proceedings of urban effectiveness evaluation.

To carry out the proposal, three key aspects must be jointly considered to monitor events that occur in the city related to accessibility issues: the advantages of sensor-based ubiquitous computing, the characteristics of wireless communication technologies, and the possibilities of cloud-based application systems. In this regard, the main novelty of this work is the integration of several strategies, paradigms, techniques, new technologies and Internet of Things solutions to build a comprehensive system that is effective in a dynamic manner.

The information generated will allow us to establish criteria for actions and urban planning concerning disability through a set of Key Accessibility Indicators (KAIs). They will be obtained by two methods: (a) by means of the inference of the patterns of movement for individuals according to their degree of disability and by comparing the routes followed in each case to conclude: for example, if people with disabilities move along the same routes as people without disabilities, or if instead they find some impediment (stairs, ramps, obstacles, etc.) along the way; (b) through the self-reporting of citizens of all accessibility deficiencies that are found anywhere when they move around the city. The remainder of the paper is organized as follows: Section 2 describes the related work on urban activities and technology used to address the analysis of the accessibility in the city; Section 3 introduces the overall system architecture for acquiring knowledge on the urban accessibility, which is explained in depth in the next sections; Section 4 explains the infrastructure needed for dynamically gathering the citizens’ locations; Section 5 describes the cloud infrastructure that supports the system; Section 6 deals with the urban accessibility information services to provide the information to stakeholders; Section 7 conducts the evaluation and discusses the results and their possibilities; and finally, Section 8 presents that study’s conclusions.

## 2. Related Work

Social awareness towards maintaining urban accessibility is growing in modern societies. The authorities have established abundant legislation at all administrative levels to create accessible and socially inclusive urban spaces. However, the methodologies used up to the present have served to eliminate some barriers in controlled urban environments but not to preserve the urban spaces over time. There are still many movement barriers, and few efforts are dedicated to analysing possible causes or elements that contribute to generating them.

The possibilities of new technologies provide innovative methods for monitoring and preserving the accessibility of urban areas. In this section, the existing methods for monitoring urban accessibility and the active technologies for performing it are reviewed. Recent and representative works are discussed to show the intensive research developed in this area. A final subsection is added to summarize the contributions to previous work.

### 2.1. Urban Accessibility Monitoring

Traditionally, the most-used method to obtain information on urban accessibility is by means of evidence, street observation and audits [10], interviews [11,12] and surveys/questionnaires [13,14,15,16,17,18,19,20,21,22,23] from authorities, disabled people and other interested groups (friends, family, etc.). Recently, new initiatives have been developed for obtaining these data from citizens themselves using new technologies, such as social network communities [24], mobile Apps and web pages [25]. In this line, preliminary works have been developed to explore the potential of communication technologies [26,27].

From the previous data, other proposals are based on mathematical and/or statistical approaches to analyse and evaluate the accessibility level of the city [21,28,29,30]. These participatory processes are certainly a challenging area of research for improving accessibility in urban areas. The following table (Table 1) summarizes various works and initiatives on this issue as a representative sample.

However, most of the proposals provide only static data. To adequately address the accessibility issue, it would be useful to know the movement habits of citizens, with or without movement disabilities, around the city anytime and anywhere. Only in this manner can the dynamic knowledge needed to verify the presence of mobility difficulties or decide corrective actions be obtained.

### 2.2. Citizen Track-and-Trace

The tracking-and-tracing of citizens is not a new topic. Knowing citizens’ traceability information for the design of cities stimulates studies and research. The main technology created to acquire citizens’ movements is the Global Positioning System (GPS). GPS can easily get a position in the world map, so it is used nowadays to orientate drivers and pedestrians since it has become quite affordable. In addition, it is now easily integrated into many devices, such as mobile phones or other ubiquitous devices [38]. With this technology, by having the exact location of people and the trajectories they have followed over time, it is possible to study their movement habits and even predict their locations at certain times of day. However, the use of GPS raises some issues to consider. One of the most important aspects to consider is that it is limited to outdoor environments. Another problem comes from its high power consumption.

An alternative to GPS technologies for sensorization of citizen movements is the use of Radio Frequency Identification (RFID) communication technology [39]. This technology consists of two types of elements: antennas or readers that emit a signal and the receivers or tags worn by the objects and users that receive it. It has also been widely used to obtain location information, as well as for object and user tracking [40,41]. In this regard, different studies have been conducted for track-and-trace indoors and outdoors. For instance, as an outdoor work, the city of London introduced a system for data collection in its transportation system, which includes buses, subways and trains [42]. This work tracked users of the transport network through payment and access cards equipped with this communication technology. With the information gathered, the level of use, number of travellers and habits of public transport in the city were determined. Likewise, it was also possible to know the stations, the breakpoints, the origin and the destination of the network travel flow. In relation to this, some other indoor systems have also been implemented to learn the movement flows of citizens [40,43]. With the results obtained, it was possible to identify route patterns of the different areas under study.

The RFID technology overcomes some of the disadvantages described for GPS, as it works indoors and reduces energy costs. However, its use for tracking does not yield exact locations because the temporal location marks are limited to the locations of antennas or signal readers. Thereby, the resolution of the trajectories followed by the users will be much lower than in the case of GPS. The scope of this technology is not comparable to GPS coverage, as it depends on the position of the antennas and the gain they use. There are two installation options with passive or active tags according to the desired range and complexity of the application. In the case of passive tags, no power supply is required for tags [44], which can be used by people who collaborate on the project.

RFID passive technologies can be presented in a multitude of objects and devices that people wear [45]. In many cases, they are integrated into standard cards made of plastic with functions of identification and access [46], credit or debit cards [47] or even paper tag on clothing [48]. Although the functions for which these elements were designed were not related to tracking people, this technology can provide high-value features for object tracking [49] and development of advanced applications of e-health [50,51] and business processes [52].

### 2.3. Findings

Some findings can be extracted after reviewing the previous work, which justify and summarize our contributions to the state-of-the-art:

- Existing methods are able to determine and quantify certain isolated problems, but they do not provide mechanisms for addressing the diversity of citizens or checking (monitoring and maintenance) its evolution over time.
- The implementation of environmental standards is already a common practice, but evaluating their effectiveness has not been established as part of the process. The static methodologies used have only been partially integrated in the participatory processes.
- Activities of localization and tracking can provide valuable information to improve the accessibility and citizen satisfaction. To perform this function, the IoT paradigm and the new communication possibilities can be used to acquire real dynamic information regarding citizens’ flows.
- A GPS system is present in the majority of new communication devices used by citizens. It can take advantage of the extensive set of mobiles connected and the installed infrastructure, but it requires the explicit collaboration of users to achieve their traceability.
- RFID technology is increasingly found embedded in devices and other portable tags accepted by the public. This makes them a good option for obtaining citizen positioning since they do not require energy consumption to function, no expensive equipment is required, and the user position can be read transparently.

The research conducted in this paper is focused on addressing the main challenge found in assessing the accessibility level in urban environments, that is, the adequacy of the infrastructures of the city and how they match with the real users’ needs. The key idea is to design a monitoring system for dynamically evaluating the effectiveness of urban accessibility. This goal will be achieved by acquiring the citizens’ movements throughout the city by using a combination of GPS technology and an IoT-based design of smart sensor networks, a distributed cloud-based architecture and the reported citizen experience through an App for smart devices. This approach allows the acquisition and analysis of the dynamic movement patterns of individuals according to their type of disability, enriched with real citizen experiences. From this information, the proposal allows smart scheduling of urban planning actions, focused not only on the removal of barriers but also on preventing such an occurrence in an active and automatic manner. In addition, the knowledge of the movement flow of citizens will allow efficient management of the resources of cities and ensure long-term sustainability in terms of enhanced service provision. This proposal is in line with other initiatives worldwide for building inclusive infrastructures in modern smart cities [53,54].

## 3. System Architecture

Once the main challenges have been pointed out and the findings of previous studies have been obtained, the following aspects must be covered to adequately address the accessibility of cities:

- Consolidating the cross-cutting nature of the accessibility from the perspective of the activities of citizen participation, that is, establishing relationships between different areas, equipment and services, different barriers to mobility and their causes.
- Providing mechanisms for developing the assessment of the current conditions of accessibility and monitoring their effectiveness over time.
- Enabling smart scheduling of urban planning actions that aim not only for the timely removal of barriers but also attempt to prevent the occurrence of barriers in a dynamic manner.
- Including citizen participation in the city development towards accessibility models.

The achievement of these aspects represents the desirable features of the required system. The proposed computational model consists of a distributed architecture that takes advantage of the new ICT technologies in a context characterized by deployment of a wireless communication infrastructure and the cloud computing paradigm in Smart City environments.

The proposed architecture is based on the experience and recent previous research works regarding distributed IoT architectures [55] and citizens’ traceability systems performed by this research group [26,27,56,57], which improve the current track-and-trace system proposals for the acquisition of locations of citizens in heterogeneous and uncontrolled environments.

This proposal combines the advantages of the previous works and integrates some of the modules tested to extend the functionalities of the entire system in several significant ways: (1) using a combination of acquisition technologies and establishing a homogeneous layer for other communication technologies; (2) designing new movement route inference methods from discrete locations; (3) improving some components of the architecture for handling large amounts of data; (4) expanding the scope for both indoor and outdoor environments; (6) especially focusing on urban accessibility issues for citizens with disabilities; and (7) integrating citizens in the urban accessibility process through user-centric mobile applications and IoT solutions.

**Figure 1:**
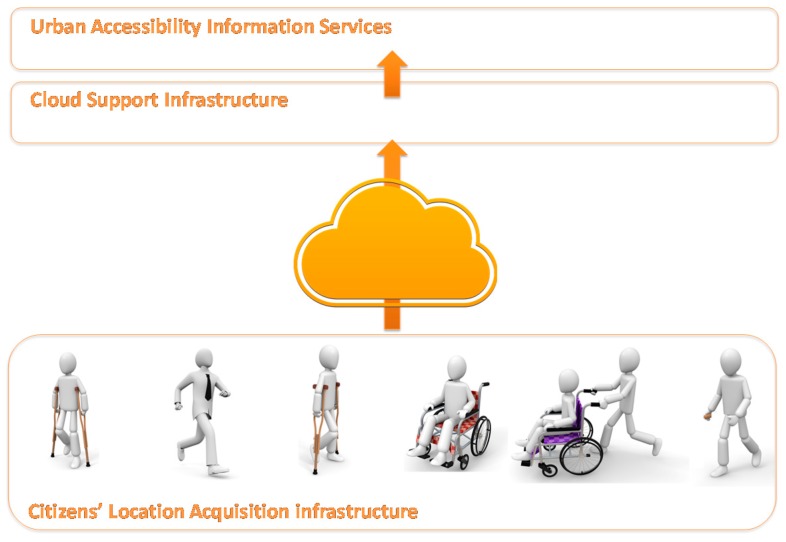
Overall architecture of the System for Accessibility Monitoring in Smart Cities.

The architecture is made up of three main layers to contain the infrastructure components and provide the services: the Citizen Location Acquisition infrastructure, Cloud Support Infrastructure, and Urban Accessibility Information Services. The basic scheme of the new architecture is shown in Figure 1. The following sections describe the design and the implementation details of each of the layers of the system.

## 4. Citizen Location Acquisition Infrastructure

The main objective of the acquisition infrastructure is to obtain the positions of citizens when they walk through the city, both in indoor and outdoor environments. This aim has the following design constraints: (1) *automation*: to be capable of acquiring and processing the individual location information in an unassisted way and transparently for citizens; (2) *scalable*: to be able to handle a variable amount of data; (3) *reliable*: to ensure the delivery and reception of each location to generate the different citizen flows; (4) *completeness*: to offer complete information about citizen movements despite the lack of certain individual locations, and their experiences in such displacements; (5): *interoperable*: to be able to obtain information from different location formats or methods, as the system is intended to take advantage of the already deployed elements incorporating them for users who might work with different protocols or formats.

The selection of the localization technology takes into account the experience acquired in the review of other works on the traceability of users and the performance and the benefits they currently offer. Two complementary technologies are used in this work to illustrate the capabilities of the system: RFID and GPS. Other wireless connections can be included in this system under the same operating principles, such as Bluetooth Low Energy (Bluetooth LE—BLE) or Near Field Communication (NFC), which are also built into most mobile phones and many consumer electronics devices. In addition, the proposed system could be valid for discrete location acquisition from users by means of widespread communication methods, such as Global System for Mobile communications (GSM) and wireless local area networking (Wi-Fi).

**Figure 2:**
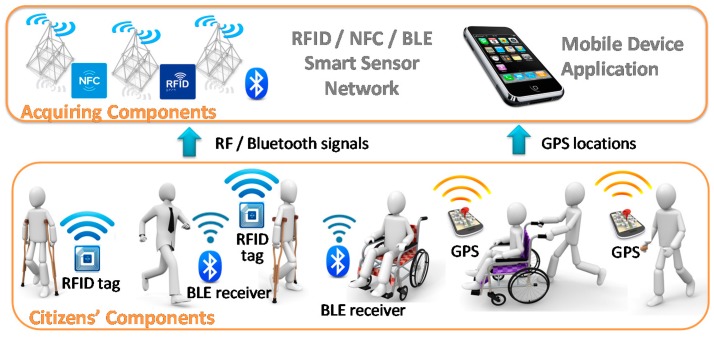
Acquisition infrastructure deployment.

The next figure (Figure 2) shows the detailed acquisition infrastructure for various communication technologies. The figure illustrates the following parts of the acquisition infrastructure: *citizen components* and *acquisition components*. These parts depend on the type of technology used in each case. In this work, RFID and GPS work together to obtain the user position. They are complementary and have some advantages over other alternatives, as described in related works, to make them suitable for the aims of this system. Obtaining positioning through GPS systems takes advantage of the wide range of phones and wireless devices that incorporate this technology already in use among the population. In this case, collaboration and user permission regarding sharing of their location are required. To facilitate this collaboration, it is necessary to guarantee the total disassociation of personal data.

This technology is scalable to the deployment of infrastructure, with the main advantage being the non-requirement of additional elements besides the satellites. Alternately, the use of RFID technologies is continuously increasing. In addition, many devices, including smartphones, currently incorporate such technology.

### 4.1. Citizen Components

The *citizen components* are the elements carried by the citizens that allow their localization. For RFID technology these elements are the Radio Frequency (RF) tags that users carry with them. For the GPS case, the component consists of the users’ smartphones. The modern devices embed a GPS module and, usually, also have an integrated RF tag and reader. Thus, in most cases, only a modern smartphone will be necessary to perform multimodal user location.

The location information acquired by both technologies must distinguish between two types of people to be useful for the model: those who have some disability and those who do not. In this manner, it will be possible to discriminate the flow of movement between the two types of citizens and draw different patterns, as well as reach conclusions. This point can only be achieved through the cooperation of citizens. Our experience in this field concludes that people with disabilities are more open to collaborate with the system since it can help them to create a more accessible city. Their family members and friends and other people who are aware of accessibility issues are also ready to participate in this project. Our previous experience has enjoyed the cooperation of all citizens whom we have asked [26,27]. In general, people are very aware of this challenge. In addition, for standard user track-and-trace, the communication technology possibilities can also get the user location and the route they follow without their express authorization, as reported in [56].

As a key aspect of the system, it is very important to maintain the privacy of these users and those who would like to participate. Therefore, this is specifically addressed by the system. As a general mandate, no personal data are gathered, only the type of disability, if any. With respect to RFID, the system tracks only the tags in objects and not people. A person can carry several tags in their clothes and accessories, and in most cases, the tag could be attached to interchangeable everyday elements, such as an umbrella or book that could be carried by different users. In addition, there are methods to preserve anonymity with RFID technology [58,59] that can be implemented in open city environments, and recent privacy-preserving mechanisms have been tailored to the characteristics of participatory sensing [60]. In this manner, the system does not know the correspondence between citizens and locations.

### 4.2. Acquisition Components

The *acquisition components* are those responsible for reading the user positions and sending them to the cloud infrastructure. This part of the architecture has been designed to cover the set of previously defined goals that are necessary to achieve the aims of the proposal. This approach covers issues that have not been taken into account in other related and previous works. Specifically, some aspects, such as environmental heterogeneity and most of the design constraints of this work, are not considered.

There are two ways to acquire the user location: by means of: (a) some communication technology for data transmission and/or (b) GPS positioning.

The communication technology requires the installation of a network of Smart Readers to capture the users’ locations, including those areas where GPS is inaccessible. These devices are mainly responsible for reading the locations automatically, as well as ensuring the delivery of such information to a central cloud server. In this part, the IoT paradigm plays an important role in providing the capabilities to match the constraint set. The design of these devices includes communication and computing features provided through an embedded system architecture, which was connected to the reader by means of some communication interface, for example, through of a Serial or Ethernet connection. This component gives it the required local intelligence and the desired functionality.

**Figure 3:**
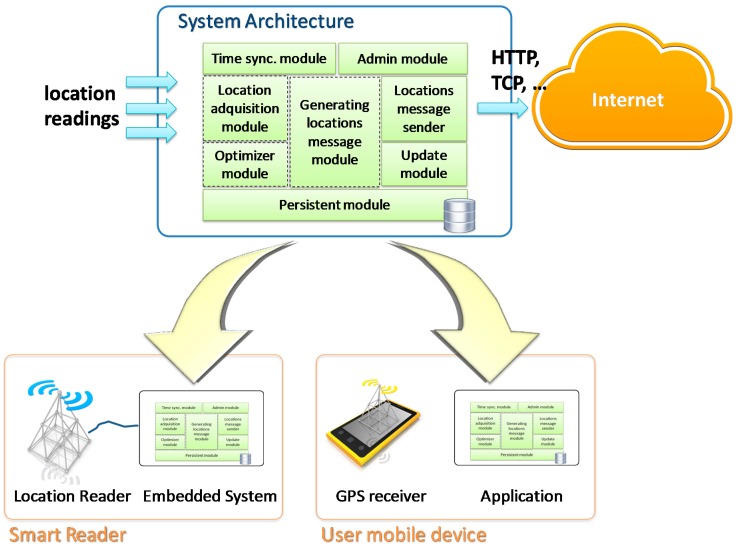
Computational architecture of the acquisition components.

The GPS user devices (such as smartphones) already include necessary communication and computational features. In this manner, the system architecture will be deployed, directly, in these devices by means of a Mobile Application or ‘App’. The main modules of the computational architecture of the acquisition components are depicted in Figure 3.

A full description of the Smart Sensor architecture for RFID was provided in our previous work [56]. In this work, the architecture has been generalized to support several methods of acquiring the user locations. Thus, different types of components can be deployed to cover a broader range of acquisition possibilities (RFID, BLE, NFC and others). The Location Acquisition Module handles the data according to the technology used and transforms it into an interoperable scheme described in the next sections.

**Figure 4:**
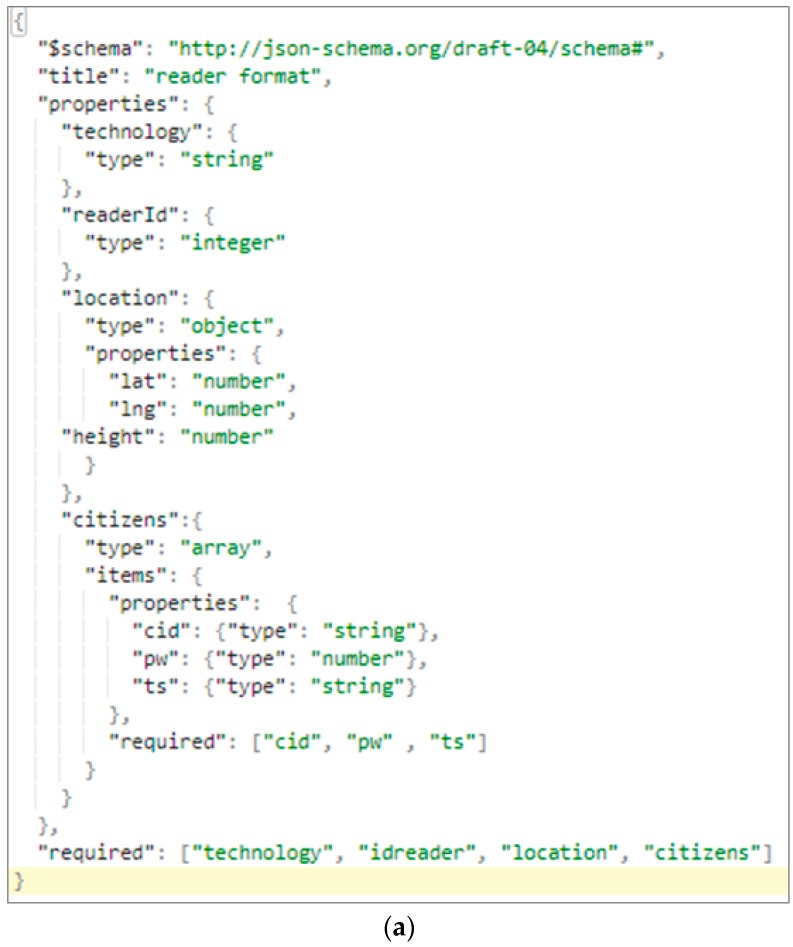

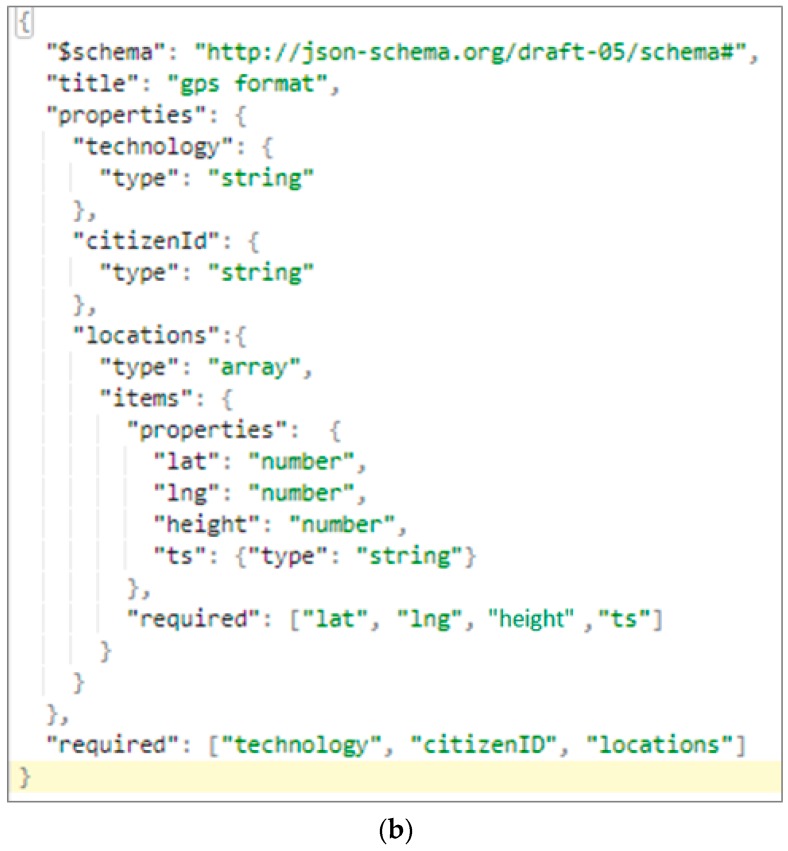
JSON locations Message formats. (**a**) RFID Message Format; (**b**) GPS Message Format.

In the previous proposal based on RFID, when a reading was received through the proximity of an RFID tag, an optimization process was called to avoid redundant readings of the same individual (*Optimizer module*). This module has been modified for the case of GPS and similar technologies. In this case, a period of periodic reading is established. In each reading of the GPS position it is compared with the previous reading. In case both readings are brought closer, based on a radius previously configured, the new reading is discarded. As a result, sending unnecessary information is avoided. Once the locations are obtained, optimized and stored, the generation of the message is performed through the *Generating locations message module*. This generation is dependent on the technology used to locate the position of citizens. In the case of technologies such as RFID, a message is generated with the position of the RFID reader but with multiple time readings of different citizens. In the case of technologies such as GPS, where readings only belong to an individual, the generated message contains multiple temporary positions of that same citizen. By default the message is generated by the JSON standard as shown in Figure 4.

In both cases, the communication channel is optimized according to the protocol used. When there are enough readings, the generated message is transmitted through the *Locations message sender* using the corresponding protocol (i.e., HTTP). In addition, a software version of this component has been developed to benefit from the presence of GPS components in many devices worn by citizens. The collected data will, in this manner, be formatted and integrated with the system.

## 5. Cloud Support Infrastructure

**Figure 5:**
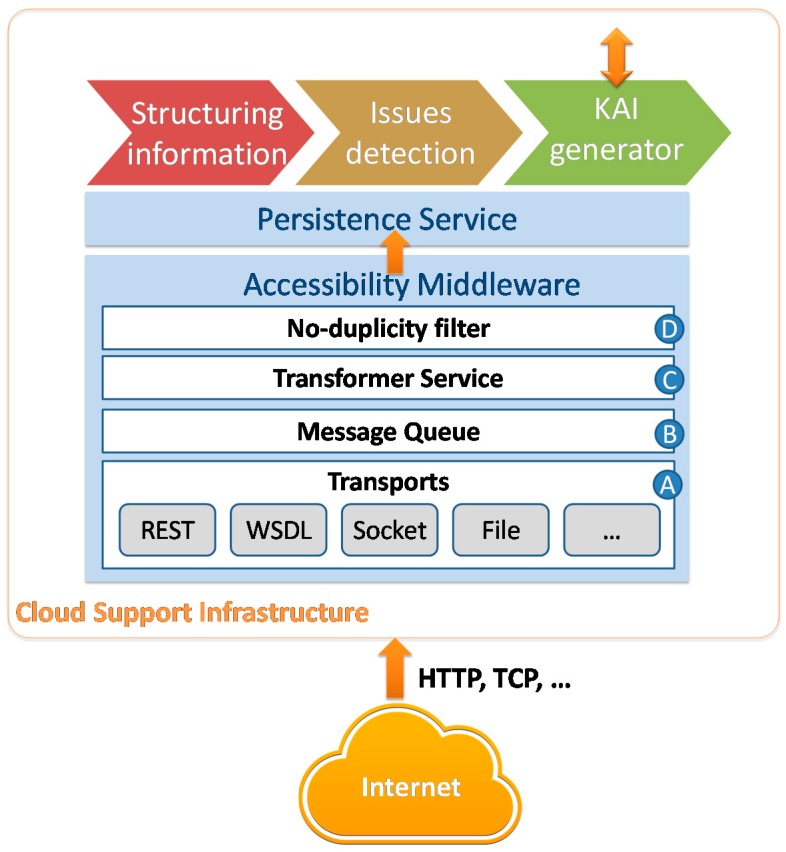
Components of the Cloud Support Infrastructure.

The *Cloud Support Infrastructure* is the centralized part of the system for the collection of all the individual locations and issues and computation for the generation of the KAIs of the city area. This infrastructure is installed on the cloud to serve for many city areas and be accessed by stakeholders. It consists of four main components: the accessibility middleware, the structuring of urban information, the accessibility issues detection, and the KAI generator for each city. Figure 5 depicts the scheme of this part of the system. The components of the system are described in the next subsections.

### 5.1. Accessibility Middleware Component

This module provides an interface for data acquisition that allows user positioning to be received from GPS devices and/or the Smart Sensors Network. This service ensures the *scalability* and *availability* of the system, *reliability* in the delivery of data, and *interoperability* with different types of sensors and technologies.

The computation needs can grow due to the number of citizens who could interact with the system and the size of the smart sensor network deployed. To provide a *scalable* system, a point-to-point message queue [61] is proposed as an input interface (Figure 5B). This method also allows a pattern guaranteed delivery model to be implemented to ensure the *reliability* of the communication of messages, converting no-transactional protocols, such as HTTP, in a transactional approach such as Java Message Service (JMS).

Another important issue to consider—when it comes to transmitting location information from smart sensors or user mobile devices—is the *interoperability* challenge due to the heterogeneity of existing customer devices on the market and the communication protocols used by them for data transmission. There are location readers with computer systems that allow communication through known and widely extended standards, however, others only include a serial communication and an embedded device, with low cost and reduced dimensions. In those cases, an infrastructure based on data model transformation patterns, data format transformation (Figure 5C) and protocol bridging [62]. The last one, in the proposal, has been implemented through the transport patters supported under the principles of the Service Oriented Architecture (SOA) and Web services technologies is proposed (Figure 5A). In this manner, the system can dynamically solve the problems of heterogeneity of the structure of the message, the data format and communication protocol used for sending the acquisition module.

When a message is received, the most important thing is store this message in a persistent system (message queue) independently from the protocol used to receive the message. This stored message will include the message itself, the acquisition technology and the transport used to receive the message. After that, the *transformer service* will consume the stored messages from the queue and will do the necessary transformation depending on the parameters included previously. Once the message has been transformed into a homogeneous format and scheme, based on JSON notation, it is stored in the information system through the persistence service. Also, a no-duplicity filter has been included to avoid message duplicity when the *ACK message* fail between one acquisition device and the cloud infrastructure (Figure 5D).

Finally, a breakdown or centralized system failure can involve the loss of location information and therefore reduce the validity of the KAI generation. To solve this problem, clustering techniques can be incorporated to achieve high *availability*. In this manner, if one of the nodes breaks down, another node in the cluster will replace it.

### 5.2. Structuring Information Component

This component performs the calculation of the possible routes followed by the citizens across the area under study. This process is performed according to three criteria: (a) completeness, (b) diversity, and (c) segmentation of routes:

(a) The completeness is related to complete information regarding the movements of citizens.
(b) The routes generated may be diverse depending on the time of the day, the day of the week and the month of the year.
(c) Another important aspect is providing separated information for citizens with disabilities and citizens without disabilities, and even the disability type.

Each route is composed by a set of single individual points read by the deployed acquisition components along the area. The single location (LOC) will be the most basic and essential entity to determine the citizen flow. This entity will be defined by the following structure:

LOC ≡ ⟨ts, id, ac, pos⟩

pos ≡ ⟨lo, la, h⟩

where

- ts: timestamp in which the reading takes place.
- id: tag or mobile device identifier that is carried by citizens.
- ac: acquisition component identifier. In the case of the application for mobile devices, this identifier is the same as id.
- pos: acquisition component position. This represents the single location of the acquisition component device determined by its longitude (lo), latitude (la) and height (h). The mobile devices are carried by the users, and the readers can be installed in a fixed manner (for example, in the street furniture) or in a mobile manner (for example, in a bus door). This scheme supports all of these approaches.

Each single route (R) is composed of a list of single locations read by the deployed acquisition components along the area under study when the citizen moves in it. That is,

R ≡ ⟨LOC_1_, LOC_2_, …, LOC_n_⟩

where for each LOC_i_ ∊ R, LOC_i_(ts) < LOC_i+1_(ts), that is, the time is increasing while the citizen goes along the route.

**Figure 6:**
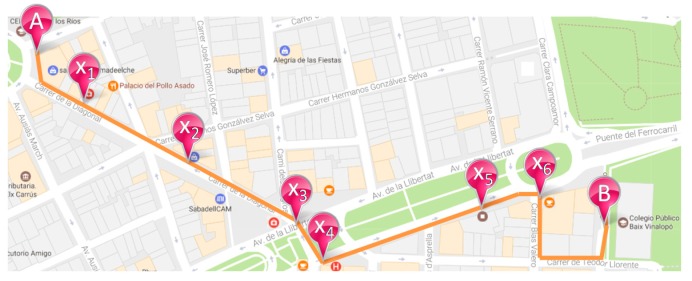
Route decomposition example.

Each single route (R_AB_) draws a path from a start point A to a destination B of the city. The points A and B are defined by the position field (pos) of LOC_1_ and LOC_n_, respectively. In addition, the routes can be decomposed on a set of subroutes where their starting and finishing points are in the initial route. Figure 6 illustrates this idea.

The following list of subroutes can be deducted from the initial route RAB depicted in Figure 6:

sub(R_AB_) = {R_Ax1_, R_Ax3_, R_x1x5_, R_x2x4_, R_x4x6_, R_x4B_, …}

where for each R_ij_, the points i and j are defined by single locations of the route R.

The inference process consists of defining the path followed by a citizen from the set of her/his discrete locations read. The first step is to complete the different routes. As defined above, the routes are a list of discrete individual points. In the case of the data read with GPS technology, the single locations are close enough together to easily infer it. However, for the other communication technologies, such as RFID or BLE, the consecutive locations can be distant in time and/or space, and an inference process is needed to build the probable path. In this case, it is useful to have a neighbouring map of the deployed acquisition component network in the city. This information will help to complete the routes from the individual locations because two consecutive reads must provide neighbour acquisition components.

**Figure 7:**
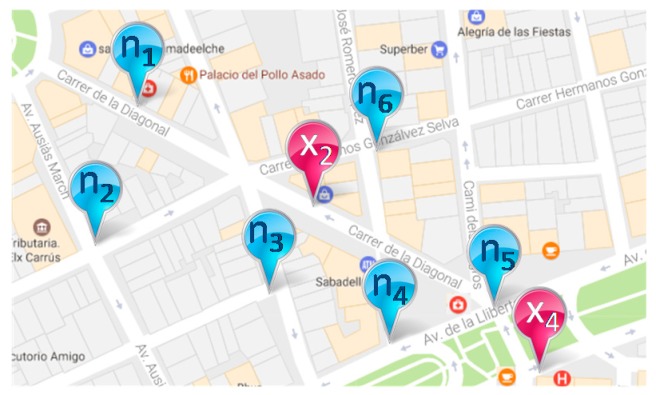
Neighbouring sensors in blue of the sensor located in X_2_.

Let SN ≡ {S_1_, S_2_, …, S_n_} be the set of acquisition components of the network. For each S_k_ ∊ SN, let n(S_k_) ⊂ SN be the neighbouring sensors of S_k_. In this manner, Figure 7 shows an outdoor example of the neighbouring sensors and how the route can be completed.

In the case shown in Figure 6, if a citizen is read by the sensor located in X_2_, and the next sensor that reads the same citizen is in X_4_, then a lack of reading takes place because X_4_ is not a neighbour of X_2_. The system can insert an automatic read by the most likely sensor and complete the route. It seems to be the sensor n_5_. Other sensors could be considered according to the time difference between the readings and the options of frequent routes. For mobile acquisition components, this method does not apply.

Once the routes have been completed, the information is ready for issue detection and querying. The queries for the diversity goal could be sent to the set of the single routes calculated, as well as all their subroutes. However, the calculation of aggregate routes provides more meaningful information about the real movement habits of the citizens. The route aggregation is performed by grouping the set of single routes that have the same starting and finishing points in a time interval, for example, all routes and subroutes that go through A and B.

The segmentation goal needs to register the accessibility issues of the area under study. To achieve this knowledge, the mentioned collaboration of the citizens with and without disabilities comes into play. The cooperating users must enter their disability type or none in the system.

### 5.3. Issue Detection Component

This component is focused on detecting the urban accessibility issues for citizens with disabilities. The proposed detection process is achieved through two methods: by means of an automatic method using the acquisition system described previously and by means of the citizen collaboration using an App for Smartphone and IoT solutions.

#### 5.3.1. Automatic Detection Method

In the first method, the basic idea consists of comparing the routes followed by citizens with and without disabilities to identify significant differences among them caused by the existence of a lack of accessibility paths. This idea was proposed in our previous works [54,56]. In this research, the methods have been improved using the advanced structured information proposed in the previous subsection.

**Figure 8:**
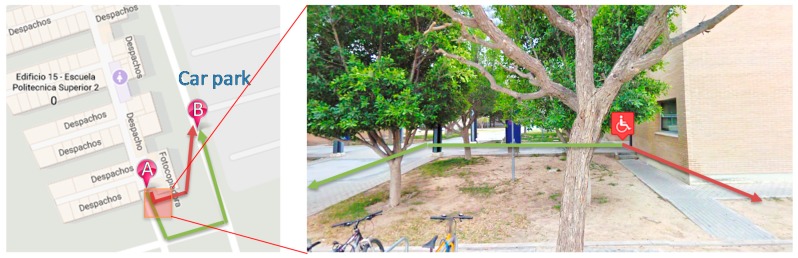
Example 1. Different routes detected due to movement disability.

The main aspect examined to automatically detect an accessibility problem is the path length. Usually, when there are differences in length, it is because the people with disabilities have to take a longer path to get to their destination. For example, Figure 8 describes this situation (coordinates: 38.387298, −0.512315). The shortest route has stairs, which prevent the passage of wheelchair users. It can be noted that the length difference between the two routes is more than twice over (57.1 m vs. 27.8 m). Then, the system raises an accessibility issue at this point.

**Figure 9:**
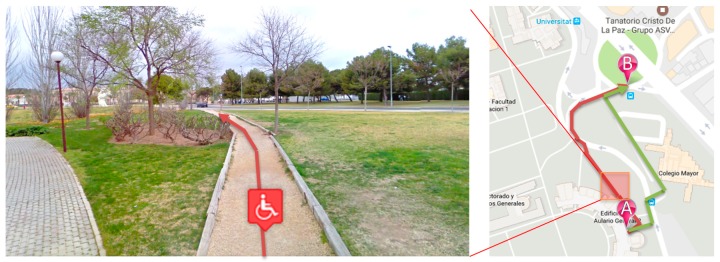
Example 2. Different routes detected due to movement disability.

Secondly, if there are no significant differences in length, the frequency of the routes followed by people with disabilities may show the problems, i.e., when such people always take the same path. For example, Figure 9 describes this situation (coordinates: 38.384863, −0.510106). In this case, both routes have the same length (252 m), but in all cases, the people with disabilities go along the green path because this way is inaccessible for wheelchairs due to the grass and the stones of the road. The system can detect an accessibility issue in this route.

Algorithm 1 describes the pseudocode of the previously proposed methods. There are some high-level functions in the method whose code is not provided, but it is easy to deduce how they work.

The selected area can be defined by rectangle coordinates, circular coordinates or a predefined area, such as a street, a building or a city district. The system must be calibrated by adjusting the threshold values and examining the false positive and negative issues detected.

Finally, a simple method consists of analysing a specific place where an accessibility action has been made to verify if this is used properly, for example, adapted toilets, ramps, lifts, etc. After a time, it can be deduced if it works correctly. Of course, once the system raises the list of issues, an in-place human monitoring is needed to check the validity of the data obtained and to identify the actions needed to overcome each issue.

**Algorithm 1.** Detection Accessibility Issues.1.Select set of aggregation routes (GR) from Selected Area2.For each route r of GR3.   let r.A be the start point of r4.   let r.B be the destination point of r5.   let dr = **dist**(r.A, r.B) be the distance from r.A to r.B6.   let sr = **dssb**(r) be the disability type of r7.   For each route s of GR8.    if r.A and r.B belong to s then9.     let ds = **dist**(s.A, s.B) be the distance from A to B following the route s10.     let ss = **dssb**(s) be the disability type of s11.     if |dr-ds| < *threshold*(sr, ss) then **raise_issue**(difference_in_length)12.     else if |**difference**(r-s)| > *threshold_d* then add(s) to ListOut(r)13.     endif14.    endif15.   endfor16.   For each type of disability d of D17.    let cr = **card**(d, r.A, r.B) be the number of routes from A to B       followed by people with the disability d18.    let cs = **card**(d, ListOut(r)) be the number of routes added in ListOut(r)       followed by people with the disability d19.    if (cr>0 and cs/cs) > *threshold_q* then **raise_issue** (difference_in_path)20.    endif21.   endfor22.endfor

#### 5.3.2. Accessibility-Issues Self-Reporting Service

This App is oriented towards obtaining the end-user experience. Its aim is to provide a user interface for mobile devices to allow citizens to add their accessibility experiences while moving around the urban environment anywhere, anytime.

The analysed works focused on the detection of urban accessibility issues are mainly based on evidence or manual surveys. With these techniques, the results obtained are from limited groups of individuals regarding a specific environment, and with more economical cost and time. In contrast, the aim of the proposed application is to change end-users into active participants in the effort to detect accessibility problems. Through this application, comments, photos or reports can be sent by citizens anonymously, and it allows information collecting on urban accessibility provided directly by the citizens. The idea behind this functionality is that the accessibility issues are notified at the moment when the user finds them. The application can report the exact location of this claim and take a photograph of it.

**Figure 10:**
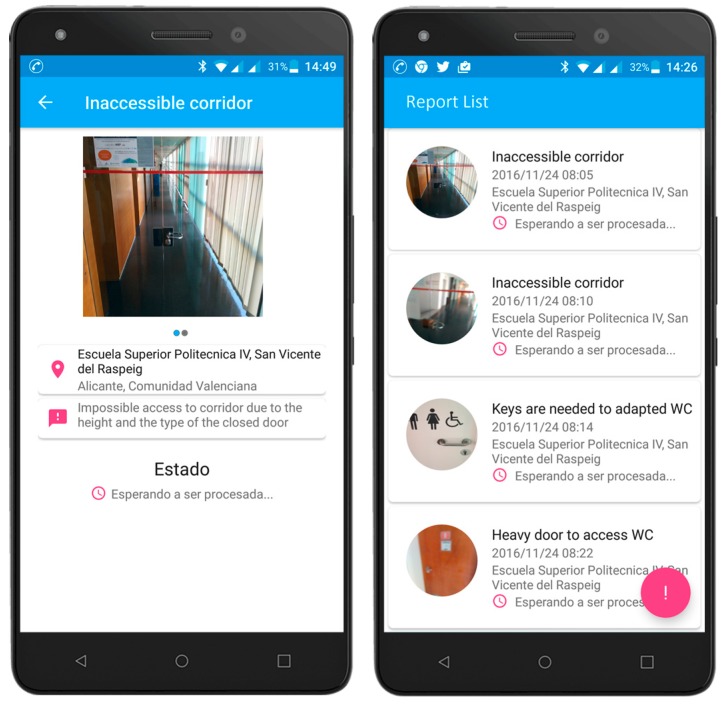
App for Accessibility Self-Reporting Issues.

The application can be installed on user mobile devices by downloading the *App*. No personal data is needed, just the disability type of the user. The next picture shows an example of the interface of this application with real data (Figure 10).

The information gathered explicitly complements that obtained through the automatic monitoring system and provides valuable information to the administrators on the status of accessibility and urban sustainability.

### 5.4. KAI Generator Component

The task of this component is to bring order and supply of different types of urban accessibility issues based on the needs and search criteria requested by stakeholders. This work is made through the generation of a set of indicators according to the type of accessibility issues detected through the two methods described previously. This converts the data into a value-added service to provide the urban accessibility information in a clear, concise and understandable manner.

The KAI defined by the system considers six cases of urban locations in this research according to their accessibility treatment and the user perceptions: (0) accessible points; (1) inefficient accessibility points; (2) automatic inaccessible points; (3) self-reported points; (4) self-reported inaccessible points; and (5) self-reported inefficient points. Table 2 lists these types. The KAIs handled by the system will be registered using the follwing structure:

KAI ≡ ⟨id, pt, pos, dt, ds, pt⟩

pt ≡ [0.5]

pos ≡ ⟨lo, la, h⟩

where

- id: #Point identifier.
- pt: Point type according to the definition shown in Table 2.
- pos: Location of the point determined by its longitude (lo), latitude (la) and height (h).
- dt: date when the point was registered, identified or detected.
- ds: description of the point, for example, accessibility policy applied, etc.
- pt: Pictures of the point.

The KAIs are generated on two time scales according to the method used to identify accessibility problems. In the case of the *automatic detection method*, described in Section 5.3.1, a specific period of time is used. Each time this period is reached, the different KAIs-of those areas from which new citizens’ movement information has been obtained—are calculated. It is a parameterized value, sufficient to collect a sample of information that determines the proposed objective.

In the second case, when the generation of KAIs is obtained through the *accessibility-issues self-reporting service* (Section 5.3.2), the generation and updating of KAIs is recalculated instantaneously. When a new citizen complaint is received, a search is performed to check if there is already a KAI in the location associated with the complaint. If it exists, the KAI information will be updated in the database, according to the accessibility rules defined in Table 2. If it does not exist, a new KAI would be generated.

Finally, an organization could directly modify a KAI through the interface offered by the *Urban Accessibility Information Service* when verifying information about that KAI or when performing an accessibility action. At that point the accessibility analysis procedure would be restarted at that point. Currently, a change in a KAI is not reflected in the different user interfaces that at that moment were visualizing an area that contains that KAI. In the future the system will update all changes of indicators in real time through an event-driven communication module.

## 6. Urban Accessibility Information Services

**Figure 11:**
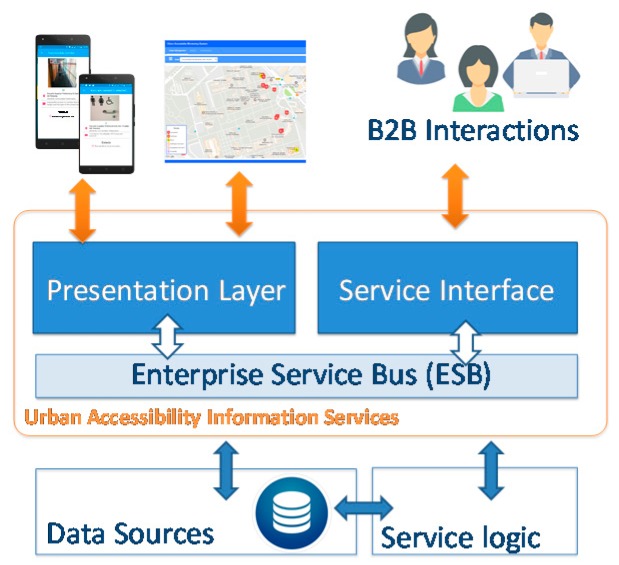
Urban Accessibility Information Service Architecture.

This layer is in charge of providing the accessibility services to the key stakeholders: citizens, city administrators, and public service provider companies, such as transportation, emergencies, etc. The provision of the services is designed according to the Service Oriented Architecture (SOA) principles paradigm [60]. The resulting information is provided in a structured manner using eXtensible Markup Language (XML) or JavaScript Object Notation (JSON) languages to enhance the interoperability. The core of the service provision architecture is an Enterprise Service Bus (ESB) platform. This is a powerful infrastructure for software integration and communication that enables interoperability, enforce security and compliance, logging and monitoring and provides agility and flexibility through of the micro-services orchestration (service logic) [61]. Figure 11 shows the overview of the information service architecture.

The results of the system should be provided following two models, with a first approach based on the paradigm of Business to Customer (B2C), oriented to offer a human-machine interface that directly allows a user to interact with the system. In this case, a presentation layer builds web or mobile interfaces for thin clients. The second approach is based on the paradigm of Business to Business (B2B), which allows consumers to connect with the service system to incorporate it as process of their organizations. This will be achieved throughout Web Services to improve the information decision task. Both protocols can define search filters to monitor a specific urban area according to the structure of the information provided.

**Figure 12:**
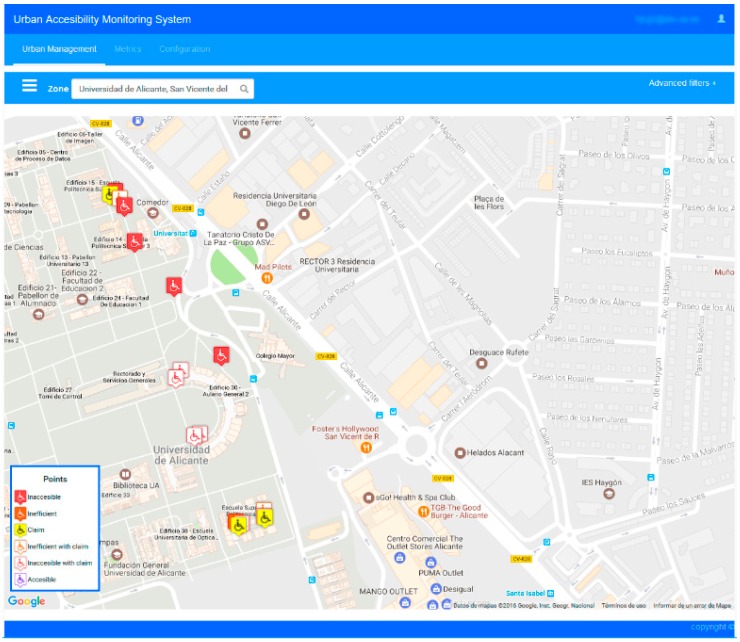
Urban Accessibility Monitoring Service Web Application Screenshot.

This interface allows surfing of the worldwide map and shows the area of interest of the user. Within this area, the application visualizes the result of the Urban Accessibility Information service and shows the KAIs corresponding to the places with accessibility issues. The user has the option of selecting one of these marks to get details about it. Figure 12 depicts an example of the web interface developed.

## 7. Evaluation and Discussion

The aim of this section is to evaluate and validate the described system and the operation of the modules and components described in the previous sections. It is expected to verify the reading technology and data processing performance in acquiring the citizens’ locations and traces along the evaluation context. In this manner, a simulated use case for people with and without disability in a real-world environment has been designed.

### 7.1. Evaluation Setup & Implementation

The technologies used to read the citizens’ locations are RFID and GPS. The development has been improved to include the new functionalities described, and both the communication and positioning technologies have been combined to acquire the locations from different sources and to support outdoor and indoor deployments.

We have implemented two acquisition prototypes to validate the proposal. First is a RFID prototype, based on our previous research described in [56]. The second is a GPS prototype through a native App for the Android system deployed over different mobile devices such as the Aquaris BQ M5.5 and E5 (BQ, Madrid, Spain).

Now, outdoor areas can be analysed by both types of technology. However, the use of RFID requires a specific installation of the acquisition components in the places of special interest, such as building entrances, bus stops, schools, etc. In contrast, the GPS technology requires the citizen’s smartphone and for the App to be installed.

**Figure 13:**
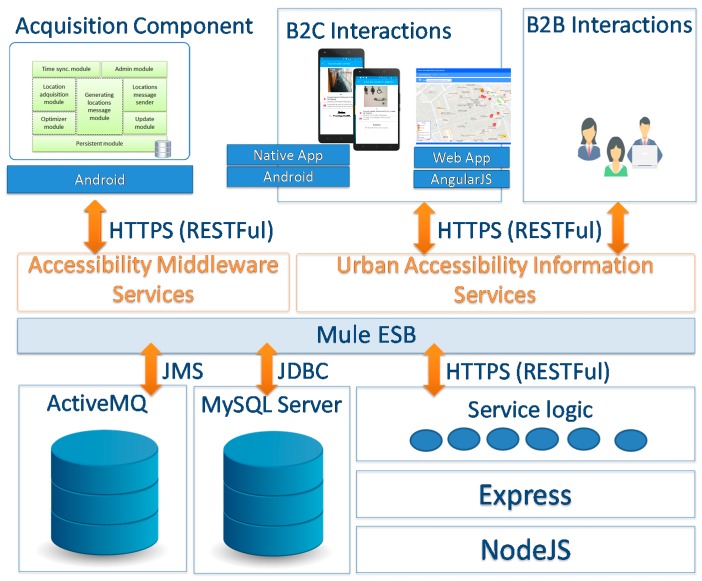
Urban accessibility monitoring service web application screenshot.

The ‘Cloud Infrastructure’ and ‘Information Services’ have been implemented using Mule ESB together with the NodeJS framework and Express Library to develop RestFul Services as a backbone element of the proposed architecture. To provide data persistence, the MySQL community version database manager has been used. In addition, ActiveMQ has been used as message service to implement the *guaranteed delivery pattern*. The presentation layer has been developed through Web technologies such as Cascading Style Sheets (CSS), HyperText Markup Language version 5 (HTML5) and JavaScript using the AngularJS framework. It provides more accessibility and usability to the final users. In addition, a web-based Geographical Information System from a third-party application (Google Maps JavaScript API v3) has been used to represent the results of the examples utilized. In addition, the ‘Accessibility-issues self-reporting service’ has been developed by means of Android Development Tools for Mobile Apps. Figure 13 shows the general prototype architecture.

### 7.2. Case Studies

In this section, two case studies are developed to explain the methods and how the system works in different scenarios. The simulation performed does not aim to be exhaustive or to collect all existing situations but rather to show the possibilities of the proposed system for detecting existing accessibility issues. In this manner, the case studies will provide a better understanding of how the accessibility of infrastructures can be assessed.

The area under study is in the campus of the University of Alicante in Spain. This site is complex enough and similar to a city but on a smaller scale that facilitates the deployment of the system. Because it has different areas (rooms, labs, small shops, restaurants, open areas, etc.) and diverse activities (academic, administrative, social, small health centres, sports, etc.), the university campus can be seen as a small city.

The evaluation was focused on analysing the daily paths of the students and lecturers across the campus (outdoors) and their movements inside of the buildings (indoors), such as the access to the classrooms, laboratories, libraries, toilets, etc.

**Figure 14:**
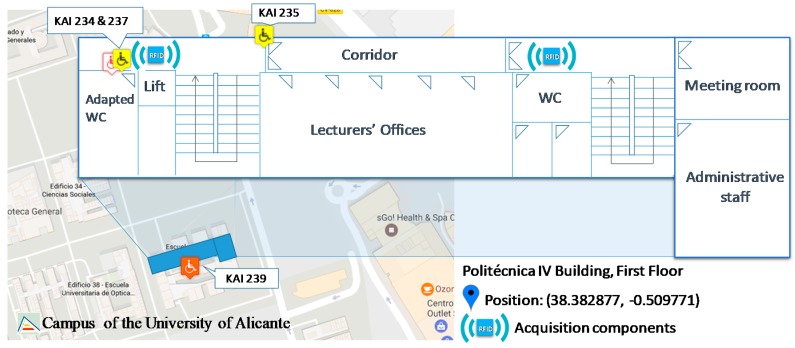
Indoor scenario and RFID-based acquisition components deployed.

Figure 14 shows the scenario used to carry out the evaluation and the infrastructure deployed in the indoor scenario. There are two RFID-based acquisition components installed in the first floor with the objective of checking the appropriate use of the WC rooms.

**Figure 15:**
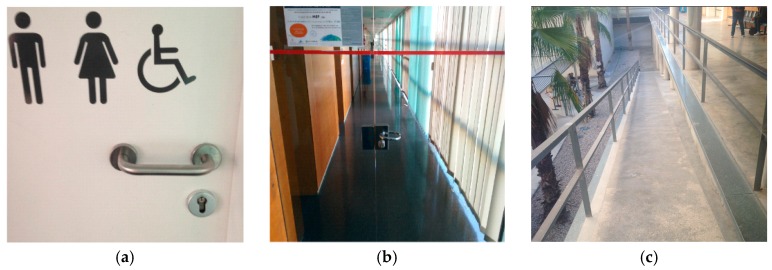
Different KAIs obtained in the validation process in Politécnica IV building. (**a**) KAIs 234 & 237; (**b**) KAI 235; (**c**) KAI 239.

After one week of operation, several KAIs were registered in the system. They come both from self-reporting application and items inferred by the system. Table 3 and Figure 15 show different KAIs identified during the evaluation period carried out in the context described above.

As shown in the previous table, the acquisition components detect that people with movement disabilities cannot use the adapted toilet. In addition, there are self-reported KAIs of the same issue. This is an example of an inefficient accessibility action since it does not meet its function. The other inefficient issue is the outdoor ramp between two floors. The GPS paths detect that it is not used by people with disabilities. A simple observation after this finding that anyone can take note of is that this ramp is too long and too steeply inclined.

**Figure 16:**
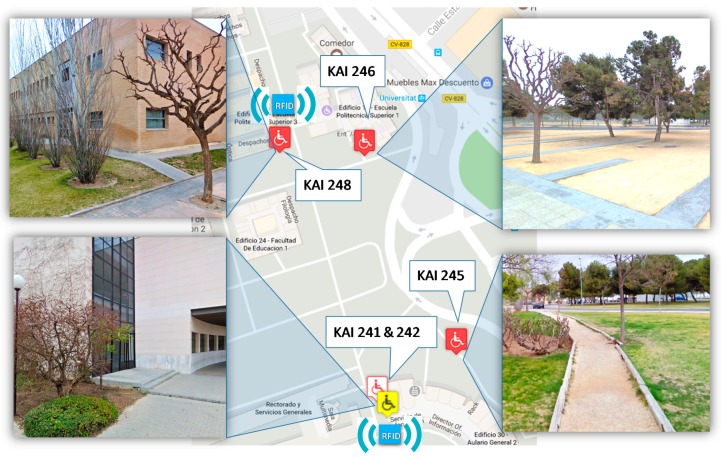
Outdoor scenario and RFID-based acquisition components deployed.

The next use case is focused on some common paths of the students and lecturers on a normal working day: from classroom building to administrative services building and lecturers’ offices. In this case, the routes are outdoors, but some acquisition components can be installed in the entrances of the buildings to trace the users’ access to them. Figure 16 and Table 4 show the area of this evaluation case and a sample of the issues registered.

Through the proposed system, different paths taken by people with and without disabilities to reach the same place or access the same building have been detected. In addition, the self-reporting contributions by users complement the information generated to build the full list of accessibility issues. The knowledge of this experience of citizens where they move in the area is very much of interest to verify the validity of the accessibility actions made. All interested users were especially helpful with this tool and, as noted in the results obtained, will serve as a source of information to prioritize some actions over others. An example of this is the multitude of inaccessible and inefficient points reported. In these cases, existing alternatives are commonly used. All these results have demonstrated the complementarity of the approaches of this proposal.

Based on the results obtained in the case studies, we have observed how the proposed system is able to automatically identify problems and deficiencies related to the accessibility of a specific area. The KAIs registered will help those responsible for managing accessibility to identify such deficiencies, prioritize them, and make decisions. We have verified that the combination of different technologies (GPS + RFID) allows for the detection of situations in heterogeneous areas in which not all the acquisition alternatives are available.

Finally, regarding the performance of the system, the computing cost of the system is variable and highly dependent on the daily information gathered. A wide deployment of the system should retrieve many locations points and routes to process. However, this is not a problem since the depth processing can be performed offline by the cloud infrastructure. In addition, the involved tasks are very parallelizable. That is, several routes can be processed in parallel by different computing elements of the cloud. In this manner, updated information can be provided to the stakeholders without delay.

## 8. Conclusions

The research presented in this paper proposes a system for evaluating the effectiveness of urban accessibility in a dynamic manner that is sustainable over time. This system provides a user-centric tool supported by technology to discover, assess and classify urban accessibility issues and generate value-added information regarding accessibility through the setting of Key Accessibility Indicators (KAI).

The proposal infers the real routes of citizens across the area under study by means of acquisition methods based on communication and positioning technologies, smart sensor networks and a distributed cloud-based architecture. In addition, the system allows acquisition of citizens’ accessibility experiences while moving around the urban environment, any place and at any time, through an App for mobile devices. From this information, the proposal allows an automatic smart scheduling of urban planning actions focused not only on the removal of barriers but also on the prevention of their occurrence.

The knowledge of movement flows of citizens with and without disability, the frequency of routes and the distribution of the main origin and destination locations will allow the efficient management of the resources of cities and ensure long-term sustainability in terms of enhanced service provision. The analysis of the identified routes together with the employed time to route them provides an accurate picture of the difficulty faced by people with disabilities, for example: urban spaces without access for people with disabilities even though these areas have high activity; urban spaces or areas highly frequented by collective with a specific type of disability; and accessible urban spaces that, temporally or with a certain frequency, become inaccessible.

## Figures and Tables

**Table 1:** Dynamic analysis of urban accessibility works clustered by methodology.

Work/Initiative		Area	Aim
*Street audits*			
	AMELIA [10]	St Albans in Hertfordshire (UK)	Increasing Accessibility
*Questionnaires and/or interviews*			
	Removing barriers Planning [22]	Spain	Accessibility diagnosis
	Public Playgrounds [14]	Malaysia	Study Disabled Children in Public Playgrounds
	Public Transportation [15]	Malaysia	Accessibility for Disabled in Public Transportation Terminal
	Public Transport [16]	Munich	Analysis of Visitor Satisfaction
	Multimodal travellers [17]	Italy	Measuring the satisfaction of multimodal travellers
	Mobility [18]	Munich	Sustainable mobility
	Welfare Urban Design [19]	Kumamoto city (Japan)	Optimal Route Finding for Wheelchair Users
	Route Navigation in Urban Spaces [13]	Northampton (UK)	Mapping for Wheelchair Users
	Welfare Town Planning Method [20]	Kumamoto central city area (Japan)	Town Planning Method for Wheelchair Users
	Commercial Complex [11]	Malaysia	Access and Accessibility Audit
	Urban areas [12]	Multi-city	Enhancing accessibility
*Mathematical and/or statistical approach*			
	Metro Systems [21]	Bangkok (Thailand)	Evaluating accessibility
	Travel Models [23]	Perth (Australia)	Evaluating accessibility
	Accessibility Planning Tools [28]	Rome (Italy)	Sustainable development
	Computer methodology [29]	Adelaide (Australia)	Evaluating urban areas for walking, cycling and transit suitability
	Accessibility in campus [30]	University of California at Santa Barbara (USA)	Measuring accessibility
	Urban transportation [31]	London (UK)	Improving the accessibility
*GIS aided*			
	Route navigation method [32]	Multi-city	Efficient Routing for disabled people
	Integral Measures of Individual Accessibility [33]	Columbus, Ohio (USA)	Measuring accessibility
	Individual accessibility revisited [34]	Multi-city	Geographical analysis of accessibility
	Time-Geographic Approach [35]	Portland metropolitan region (USA)	Geovisualization of Human Activity Patterns
	Sustainable Transport [36]	London (UK)	Transport Accessibility Analysis
	Accessibility analysis [37]	Auckland region (New Zealand)	Accessibility via public analysis
*Communication technology aided*			
	RFID based [26,27]	Alicante (Spain)	Analysis of Accessibility of Disabled People
*Self-reporting and/or social network interaction*			
	mPASS [24]	Multi city	Identify accessibility issues
	Wheelmap project [25]	Multi city	Identify accessibility issues

**Table 2:** Type of urban locations according to accessibility treatment.

#KAI		Description	Example
(0) Accessible	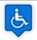	Urban location where accessibility policies were applied.	Wheelchair ramp.
(1) Inefficient	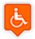	Urban location where accessibility policies were applied that are not operating properly.	Wheelchair ramp with too big slope.
(2) Inaccessible	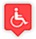	Urban location not suitable for people with mobility problems.	Entrance stairs.
(3) Self-reported	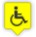	Urban location reported by users.	Entrance stairs.
(4) Self-reported inaccessible	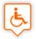	Urban location not suitable for people with mobility problems reported by users.	Entrance stairs.
(5) Self-reported inefficient	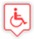	Urban location where accessibility policies were applied that are not operating properly reported by users.	Wheelchair ramp with too big slope.

**Table 3:** Different KAIs obtained in the validation process in Politécnica IV building.

Test 1. Student Movements in EPS IV Building			
KAI #	Description	KAI	Source
KAI 234	Inaccessible WC in module 3 of Politécnica IV building, first floor.	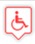	Self-reported & Inefficient inferred
KAI 235	Impossible access to lecturers’ offices due to the height and the type of the closed door.	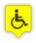	Self-reported
KAI 237	WC adapted for disabled is closed. Keys are required for entry.	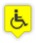	Self-reported
KAI 239	Ramp of access to different classrooms of theory and laboratories too long and too slope.	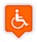	Inefficient inferred

**Table 4:** Different KAIs obtained in the validation process along some common paths.

Test 2. Common Path among Some Buildings of the Campus			
KAI #	Description	KAI	Source
KAI 241	Inaccessible curb at the entrance of the classroom building.	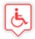	Self-reported & Inaccessible inferred
KAI 242	A very heavy door prevents access to the classroom building.	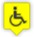	Self-reported
KAI 245	Road with stones prevents the use of wheelchairs.	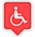	Inaccessible inferred
KAI 246	Sand Shortcut to administrative services building prevents the use of wheelchairs.	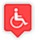	Inefficient inferred
KAI 248	The side access of the main door in EPS III building has an insurmountable curb.	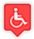	Inefficient inferred
